# Dissecting the Gut Microbiota-metabolite-coronary Atherosclerosis Axis: Evidence from Two-sample Mendelian Randomization

**DOI:** 10.2174/0118715303456504260611042850

**Published:** 2026-07-21

**Authors:** Qitian Sun, Zeyu Zhou, Yu Gao

**Affiliations:** 1 Department of Endocrinology, Affiliated Hospital of Chengde Medical University, Chengde, Hebei, China;; 2 Department of Clinical Laboratory, Affiliated Hospital of Chengde Medical University, Chengde, Hebei Province, China;; 3 Hebei Key Laboratory of Panvascular Diseases, Chengde, Hebei Province, China

**Keywords:** Gut microbiota, plasma metabolites, coronary atherosclerosis, Mendelian randomization, causal mediation, metabolic pathways

## Abstract

**Introduction:**

Coronary Atherosclerosis (CAS) is a complex disease influenced by host genes, the gut microbiota, and circulating metabolites. Causal relationships and mediating effects among these factors have not yet been clarified.

**Methods:**

A two-sample Mendelian Randomization (MR) study was conducted using genome-wide association study data from FinnGen, OpenGWAS, and Canadian Longitudinal Study on Aging. A total of 473 genetic instruments for gut microbial traits and 1,400 for plasma metabolites were selected. We applied bidirectional Mendelian randomization, sensitivity tests, and two-step mediation analysis to assess causal effects and identify metabolic mediators.

**Results:**

18 gut microbiota taxa and 36 metabolites that are associated with coronary atherosclerosis. Protective taxa included *Genus Roseibacillus* and *Genus Negativibacillus*; *Genus Geobacter C* and *Species Lawsonibacter sp000492175* were identified as risk. Inversely, bile acid derivatives, such as deoxycholic acid 12-sulfate, were negatively correlated, whereas guanidinoacetate was positively correlated. Mediation analysis found glutamine degradant, sphingomyelin, and 3β-hydroxy-5-cholestenoate as partially mediated by the microbial effect, with mediation proportions ranging from -30.1% to 15.8%.

**Discussion:**

These findings provide genetic evidence for the involvement of the gut microbiota in the development of coronary atherosclerosis, which may occur through metabolic pathways. Beneficial microbial taxa may be preferentially sustained by amino acid metabolism and anti-inflammatory mediators, whereas pathogenic taxa tend to proliferate under conditions characterized by lipid and sterol metabolic dysregulation.

**Conclusion:**

This study emphasizes the potential central axis of gut microbiota-plasma metabolites-CAS and identifies microbial and plasma metabolite candidates that may serve as targets for early prevention and intervention of CAS. These findings provide new insights into the molecular mechanisms underlying the interactions between gut microbiota and plasma metabolites in the development and progression of CAS, and offer a reference for future exploration of precision medicine strategies targeting CAS.

## 1 INTRODUCTION

Cardiovascular Diseases (CVDs) represent a leading cause of morbidity and mortality worldwide, significantly impairing quality of life. It is estimated that y 17.9 million individuals die from CVDs each year [1]. Atherosclerosis is generally considered the fundamental lesion underlying most cardiovascular events and affects the systemic vasculature, with the coronary arteries most frequently involved. The aging of the population is becoming increasingly serious, and the burden on the public health system is growing day by day [2]. Atherosclerosis is a disease with multiple etiologies and consequences, and therefore, it has always been of interest, and its study has been conducted at both the fundamental scientific and applied clinical levels. CAS, as a type of cardiovascular disease, is a chronic and progressive systemic disorder, and most patients first have clinical manifestations when the disease progresses to acute coronary syndrome, which is why it is called the “silent killer”. It begins with endothelial dysfunction, and platelets, leukocytes, and other cells adhere and aggregate, then release large numbers of inflammatory and thrombotic factors.

In recent years, multi-omics approaches have brought new insight into the molecular mechanisms of coronary atherosclerosis. For example, 161 patients with coronary heart disease and 40 normal controls underwent a multi-omics analysis, and it was found that the composition of the gut microbiota and the metabolomic profiles related to disease severity differed. Another study found molecular features that could predict high-risk atherosclerotic plaques and adverse cardiovascular events [3]. However, whether circulating metabolites mediate between gut microbiota and coronary atherosclerosis is also insufficiently studied.

A large body of evidence shows that gut microbiota plays an important role in the development and progression of various metabolic cardiovascular diseases [4], including hypertension, heart failure, atherosclerosis, and coronary artery disease, especially in relation to coronary atherosclerosis [5]. As a critical interface between the host and the environment, gut microbiota influences cardiovascular homeostasis through multiple mechanisms, including regulation of energy metabolism, immune-inflammatory responses, and signaling molecule production. In recent years, probiotics and other microbiome-targeted interventions have emerged as possible adjunctive approaches to slow or attenuate coronary atherosclerosis [6].

It is worth noting that the effects of gut microbiota on the host are mainly mediated by their metabolic products, such as short-chain fatty acids, bile acid derivatives, and trimethylamine N-oxide [7-9]. Once these metabolites enter the circulation, they participate in inflammation regulation, lipid metabolism, and the maintenance of endothelial cell function, thereby determining whether and to what degree coronary atherosclerosis will occur and progress [10]. Multi-omics studies have suggested that changes in the gut microbiota are often associated with substantial alterations in plasma metabolomic profiles, suggesting causal relationships [11]. However, direct evidence for circulating metabolites being mediators is still missing.

In terms of method, conventional observational studies have found many links between gut microbiota, metabolites, and coronary atherosclerosis; however, these conclusions are all incomplete and unable to separate cause and effect. By using genetic variants as instruments, Mendelian Randomization (MR) can reduce confounding and address reverse causality, making it a powerful tool for disentangling the complex causal relationships between exposures and outcomes [12]. Applying MR to investigate the relationships among gut microbiota, plasma metabolites, and coronary atherosclerosis may help to partially overcome the limitations of conventional observational studies, enabling a more robust assessment of the potential causal pathways through which microbial and metabolic factors influence disease development, and providing a reference for the identification of potential etiological biomarkers and therapeutic targets [13]. In the context of precision medicine, integrating multi-omics data—such as the microbiome, metabolome, and clinical phenotypes—offers a novel perspective for cardiovascular research, potentially facilitating a more comprehensive understanding of disease mechanisms and supporting individualized early risk assessment and intervention strategies. Clarifying the potential causal roles of gut microbiota and plasma metabolites in coronary atherosclerosis could provide insights for mechanistic studies, the identification of molecular biomarkers, and the development of targeted prevention and personalized treatment approaches [14].

## 2 MATERIALS AND METHODS

### 2.1 Study Design

The study's design scheme is shown in Fig. (**1**). Within this framework, an integrative multi-omics strategy was applied to capture the complex interactions between host genetics, microbial composition, and metabolic profiles, thereby providing a systems-level perspective for precision cardiovascular risk evaluation. This research used an MR framework to investigate the potential causal relationships among gut microbiota, circulating plasma metabolites, and coronary atherosclerosis, and to determine whether these factors may act as mediators. The procedure involved: first, evaluating the causal impact of gut microbiota on coronary atherosclerosis; then, examining the effect of plasma metabolites on coronary atherosclerosis. Bidirectional MR analyses were performed to determine the directionality of causality. Next, the causal influence of gut microbiota on plasma metabolites was explored to identify potential metabolic mediation pathways. Finally, a two-step MR method was used to assess the mediating role of plasma metabolites in the link between gut microbiota and coronary atherosclerosis.

Additionally, Fig. (**1**) also depicts the three essential assumptions for using Single-Nucleotide Polymorphisms (SNPs) as Instrumental Variables (IVs): (i) the IVs are significantly associated with the exposure; (ii) known or unknown confounders do not influence the IVs; (iii) the IVs affect the outcome exclusively through the exposure [15]. All data used in this study were obtained from the FinnGen and OpenGWAS public databases and had been de-identified before upload to protect individual privacy. The summary statistics employed are publicly available and ethically compliant. Notably, the data for coronary atherosclerosis, gut microbiota, and plasma metabolites were derived from distinct cohorts, ensuring no sample overlap [16].

### 2.2 Data Sources

The gut microbiota Genome-Wide Association Study (GWAS) data used in this study were sourced from the most recent gut microbiome genomics project, encompassing 473 microbial traits. These data are publicly available on the GWAS platform (https://gwas.mrcieu.ac.uk/, accessed on January 15, 2025) under summary statistics IDs GCST90032172-GCST90032644, based on genome-wide genotyping of 5,959 Finnish individuals [17]. Plasma metabolite data were obtained from the Canadian Longitudinal Study on Aging (CLSA) [18], including 7,824 European adults from two population-based studies, with genome-wide genotyping and GWAS of 1,091 circulating metabolites and 309 metabolite ratios. The outcome GWAS summary data were obtained from the FinnGen R12 database (https://www.finngen.fi/en/access_results, accessed on January 15, 2025), which includes genome-wide genotyping of 500,348 individuals of European ancestry [19], comprising 63,307 coronary atherosclerosis cases and 416,171 controls. All data are high-quality, publicly accessible summary statistics that provide a robust foundation for MR analyses [20].

### 2.3 Genetic Instrumental Variables (IVs) Selection

For IV selection, SNPs associated with gut microbiota traits were first identified. Given the generally weak effect sizes of microbiota-associated SNPs, a minimum of 3 relevant SNPs per trait was required to enhance representativeness and analytical stability, with a significance threshold of *p < 5×10^−6^* [21]. For plasma metabolites, SNPs were chosen based on a genome-wide significance threshold of *p < 5×10^−8^.* SNPs were pruned with r^2^ < 0.001 to avoid linkage disequilibrium and separated by at least 10,000 kb [22].

SNP data for exposures and outcomes were obtained from the IEU OpenGWAS and FinnGen databases, and palindromic SNPs (A/T or G/C) were excluded to prevent bias due to ambiguous alleles. After these procedures, the remaining SNPs were considered valid IVs. Bonferroni correction was applied to control for multiple testing, yielding significance thresholds of 6.84 × 10^−5^ (0.05/731) for immune cell phenotypes and 1.05 × 10^−4^ (0.05/473) for gut microbiota. Results with *p*-values between 0.05 and the adjusted threshold were considered suggestive [23].

For each SNP, key data such as chromosomal position, Effect Allele (EA), Other Allele (OA), Effect Allele Frequency (EAF), effect size (β), Standard Error (SE), and P-value were gathered. The proportion of variance explained (R^2^) and F-statistics were calculated to evaluate instrument strength using the following formulas: R^2^ = 2 × EAF × (1 - EAF) × β^2^ / [2 × EAF × (1 - EAF) × β^2^ + 2 × EAF × (1 - EAF) × N × SE^2^], where N is the adequate sample size; F = R^2^ × (N - 2) / (1 - R^2^). Weak IVs with F < 10 were excluded to ensure robustness. Odds Ratios (ORs) were used to quantify the association between IVs and binary outcomes, reflecting the potential impact of exposures on the risk of the outcome (Supplementary **1**) [24].

### 2.4 Statistical Analysis

All analyses were carried out using R (v4.4.1) and the packages TwoSampleMR (v0.6.8), MRPRESSO (v1.0). Five methods of MR were used: inverse-variance weighting (IVW), weighted median, model alone, weighted model, and regression-MR (regression-ero-gerton), where ivw was used primarily. Results were considered robust if IVW betas were statistically significant (*p* < 0.05) and other methods yielded β values in the same direction (though not significant). ORs and 95% CI for coronary atherosclerosis were estimated; *p* < 0.05 was considered statistically significant.

Heterogeneity was evaluated using Cochran’s Q test (*p* < 0.05 indicates significant heterogeneity). Horizontal pleiotropy was assessed *via* MR-PRESSO and MR-Egger intercept tests (*p* > 0.05 suggests no pleiotropy). A leave-one-out sensitivity analysis was performed to examine the influence of individual SNPs on causal estimates.

Mediation analyses were conducted using a two-step MR approach. First, the total effect of gut microbiota on coronary atherosclerosis (β_all) was estimated. Next, the effects of gut microbiota on plasma metabolites (β_1_) and plasma metabolites on coronary atherosclerosis (β_2_) were calculated. The mediated effect was derived as β_1_ × β_2_, and the direct effect was calculated as the total effect minus the mediated effect (β_all - β_1_β_2_). A *p-value* < 0.05 indicates a significant mediation effect (Fig. **2**).

## 3 RESULTS

### 3.1 Causal Relationship Between Gut Microbiota and Coronary Atherosclerosis

Mendelian randomization analyses were conducted using the IVW method to assess potential causal links between 473 gut microbiota traits and coronary atherosclerosis, with heterogeneity and pleiotropy tests to ensure robustness.

Using MR, we identified 18 key gut microbiota taxa, including one family, four genera, and 13 species, that showed significant causal associations with CAS. Notably, *Genus Geobacter C* (OR = 1.3623; 95% CI: 1.0661-1.7409; *p* = 0.0135), *Species Lawsonibacter sp000492175* (OR = 1.3511; 95% CI: 1.0347-1.7642; *p* = 0.0271), and *Species Bin127* (OR = 1.3008; 95% CI: 1.0258-1.6495; *p* = 0.0300) were positively associated with increased coronary atherosclerosis risk. Conversely, *Genus Roseibacillus* (OR = 0.6435; 95% CI: 0.4751-0.8716; *p* = 0.0044), *Species Hungatella sp900155545* (OR = 0.8195; 95% CI: 0.7033-0.9549; *p* = 0.0107), *Species Eubacterium callanderi* (OR = 0.8279; 95% CI: 0.7159-0.9574; *p* = 0.0109), and *Genus Negativibacillus* (OR = 0.8331; 95% CI: 0.7636-0.9089; *p* < 0.001) were inversely associated with coronary atherosclerosis. Cochran's Q tests for the selected taxa yielded *P-values* > 0.05, indicating no significant heterogeneity. MR-Egger intercept tests showed no statistical significance, suggesting the absence of horizontal pleiotropy. Leave-one-out sensitivity analyses confirmed the robustness of causal estimates, as the exclusion of individual SNPs did not alter the results (Supplementary Tables **S1** and **S2**). Fig. (**3**) presents the IVW-based forest plot of these 18 key gut microbiota traits as exposures and coronary atherosclerosis as the outcome. Results from four other MR methods were consistent with the IVW approach, further validating the robustness of the findings (Supplementary Figure **1**).

Bidirectional MR analyses were also performed, showing that coronary atherosclerosis did not significantly affect the 18 gut microbiota taxa (all *p* > 0.05) (Supplementary Table **S3**).

### 3.2 Causal Relationship Between Plasma Metabolites and Coronary Atherosclerosis

Two-sample MR identified 36 plasma metabolites significantly associated with coronary atherosclerosis from 1,400 metabolites, including 32 metabolite levels and 4 metabolite ratios. Specifically, 23 metabolites or ratios (*e.g*., Octadecanedioate, X-21286, Creatine-to-carnitine ratio, X-26111, Deoxycholic acid 12-sulfate, Deoxycholic acid glucuronide) were negatively associated with coronary atherosclerosis, with ORs ranging from 0.8449 to 0.9695, suggesting that increased levels may reduce disease risk.

Four ratio-type metabolites (*e.g*., Creatine-to-carnitine ratio, Glycine-to-serine ratio, Paraxanthine-to-5-acetylamino-6-formylamino-3-methyluracil ratio) were significantly associated with coronary atherosclerosis, with ORs ranging from 0.8605 to 1.0311, indicating potential regulatory roles of metabolite ratios in disease development.

Additionally, 13 metabolites or ratios (*e.g*., Glycohyocholate, Glutamine degradant, X-18888, Guanidinoacetate) were positively linked to coronary atherosclerosis (ORs: 1.0311-1.1734), suggesting that elevated levels may increase the risk of the disease. These results imply potential causal roles of specific plasma metabolites in coronary atherosclerosis, laying the groundwork for further mediation analyses (Fig. **4**). Four other MR methods validated the IVW findings, confirming their robustness (Supplementary Figure **2**). Sensitivity analyses revealed no significant heterogeneity or horizontal pleiotropy (Supplementary Tables **S4** and **S5**).

Bidirectional MR analyses indicated that coronary atherosclerosis had significant causal effects on only X-21467, X-26111, 3-methylglutaconate, and X-21471 levels, whereas the remaining metabolites or ratios showed no significant reverse causation (all *P > 0.05*) (Supplementary Table **S6**).

### 3.3 Plasma Metabolites Mediate the Effect of Gut Microbiota on Coronary Atherosclerosis

Phenotypes with potential reverse causation were excluded to ensure the validity of causal inference. A two-step MR approach was applied to assess potential mediation using the selected gut microbiota and plasma metabolites. Specifically, 18 gut microbiota traits were used as exposures, and 32 plasma metabolites or ratios were used as outcomes in MR analyses examining the effects of gut microbiota on plasma metabolites (Fig. **5**). Thirteen gut microbiota taxa were significantly associated with 16 plasma metabolites or ratios. For example, *Genus Roseibacillus* was negatively associated with Methionine sulfone levels (OR = 0.5376; 95% CI: 0.3025-0.9555; *p* = 0.0344) but positively associated with 1-palmitoyl-2-oleoyl-GPI (16:0/18:1) levels (OR = 2.4387; 95% CI: 1.3263-4.4842; *p* = 0.0041). Heterogeneity and pleiotropy assessments (Supplementary Tables **S7** and **S8**) supported the robustness of these findings.

### 3.4 Mediation Analysis of Gut Microbiota, Plasma Metabolites, and Coronary Atherosclerosis

Mediation analyses were conducted based on identified key metabolites regulated by gut microbiota. The effect of gut microbiota on plasma metabolites (β_1_) and the mediating effect of metabolites on coronary atherosclerosis (β_2_) were sequentially estimated. Three plasma Metabolites significantly mediate the relationship between gut microbiota and coronary atherosclerosis (Table **1**). *Species Bin127* abundance exerted a partial mediating effect *via* glutamine degradant (β = -0.0791; 95% CI: -0.139 to -0.0192), accounting for -30.1% of the total effect (95% CI: -52.8% to -7.28%; *P = 0.0097*).

Interestingly, *Family Thermococcaceae* abundance mediated CAS *via* sphingomyelin (d17:1/16:0, d18:1/15:0, d16:1/17:0) levels (β = 0.0359; 95% CI: 0.000642-0.0712, 14.1% of total effect, 95% CI: 0.252%-28.0%; *p* = 0.0460) and 3β-hydroxy-5-cholestenoate levels (β = 0.0403; 95% CI: 9.79×10^−5^-0.0805, 15.8% of total effect, 95% CI: 0.0384%-31.6%; *p* = 0.0494). These results suggest that gut microbiota, such as Bin127 and Thermococcaceae, may influence coronary atherosclerosis *via* distinct metabolic pathways, with glutamine degradation, sphingomyelin, and 3β-hydroxy-5-cholestenoate serving as key mediators in the gut microbiota-CAS axis.

## 4 DISCUSSION

This large-scale Mendelian randomization study provides preliminary genetic evidence suggesting that gut microbiota may influence CAS risk *via* specific plasma metabolites. We identified 18 gut microbial taxa and 36 plasma metabolites or their ratios that are potentially causally associated with CAS. Notably, two-step MR mediation analysis indicated that the effects of certain microbes, such as Species Bin127 and the family Thermococcaceae, on CAS may be partially mediated through the regulation of key circulating metabolites, including glutamine degradation products, sphingomyelins, and 3β-hydroxy-5-cholestenoate. These findings suggest that the “gut microbiota-metabolite” axis may play a role in CAS development and provide a reference for future mechanistic studies and potential intervention strategies.

Initial MR analysis confirmed that Gut Microbiota were Causal Upstream risk factors for CAS. Protective effects observed in the genera Roseibacillus and Negativibacillus are consistent with previous studies showing that beneficial microbes participate in the inflammatory process through metabolites, such as short-chain fatty acids [25]. Conversely, the risk-related effects of Genus Geobacter C suggest that it may be involved in inducing inflammation, which is consistent with previous studies [26]. Bidirectional MR analyses did not detect significant causality from CAS to these taxa, further supporting the inference of unidirectional causality.

Plasma metabolite analysis identified another 36 causal mediators. CAS had causal effects on only 4 metabolites with any significant strength, indicating that most metabolites likely operate upstream rather than downstream of disease. These metabolites may also serve as a potential therapeutic target. For instance, the negative correlation of deoxycholic acid 12-sulfate indicates that it may have a protective effect, possibly through the enhancement of bile acid detoxification and excretion; Guanidinoacetate is positively correlated with it, which implies that it is involved in the body's energy metabolism, and the dysregulation of vascular oxidative stress increases the risk of CAS [27, 28]. Creatine-to-carnitine ratio and other metabolite ratios also underscore the importance of metabolic balance for CAS risk.

One of the main contributions of this study is the ability to quantitatively assess mediation effects and explore potential mechanisms. Although *Species Bin127* showed an overall OR greater than 1 for CAS, its indirect effect through glutamine-degrading factors was negative (mediation effect = -0.0791; mediation proportion = -30.1%), suggesting that this metabolic pathway may contribute to Bin127's overall effect on CAS. Specifically, Bin127 may inhibit the levels of glutamine-degrading factors (OR < 1), which are themselves positively associated with CAS risk (OR > 1). Therefore, Bin127 may indirectly modulate glutamine metabolism, thereby affecting CAS risk. Glutamine serves as an important energy substrate for immune and endothelial cells [29], and Bin127 may reduce excessive glutamine degradation, promote the synthesis of antioxidants such as glutathione, and maintain immune cells in a relatively quiescent state, thereby potentially influencing vascular inflammation. Notably, previous studies have shown that the PON1 gene polymorphisms at positions 55 (leucine, L allele) and 192 (glutamine, Q allele) individually have no significant effect on carotid intima-Media Thickness (IMT), but the combined LL/QQ genotype is associated with increased IMT in patients with familial hypercholesterolemia. Considering that PON1 activity is closely linked to glutamine metabolism and antioxidant function, these findings suggest that Bin127-mediated modulation of glutamine metabolism may influence oxidative stress and endothelial function, thereby contributing to atherosclerosis [30]. Taken together, these results indicate that the probiotic Bin127 and its metabolic traits, in combination with genetic factors, may provide new insights into potential strategies for CAS prevention.

In contrast, the family Thermococcaceae may be associated with CAS risk through two potential independent positive pathways, involving sphingomyelins (*e.g*., d17:1/16:0) and 3β-hydroxy-5-cholestenoate, which account for a portion of the overall risk (mediation proportions: 14.1% and 15.8%, respectively). Sphingomyelin is an important component of cell membranes and lipoproteins, such as Low-Density Lipoprotein (LDL), and elevated levels may promote lipoprotein retention in the vascular wall and activate inflammatory responses [31]. Studies have indicated that under inflammatory conditions, sphingomyelins and their derivative sphingosine can modulate monocyte tissue factor-mediated coagulation, suggesting that sphingomyelin may play a role in the interplay between lipid metabolism and inflammation-related immune responses [32]. Further biochemical investigations have shown that desialylated LDL exhibits reduced sphingomyelin content, altered cholesterol and sterol composition, and enhanced lipid accumulation in vascular cells [33]. Moreover, upon uptake of Oxidized LDL (Ox-LDL) by macrophages, sphingomyelin and Unesterified Cholesterol (UC) significantly accumulate in lysosomes, and 7-hydroxycholesterol (7-KC) further promotes this process by inhibiting lysosomal sphingomyelinase. These UC-SM lysosomal particles are present in atherosclerotic lesions, suggesting that dysregulated lysosomal sphingomyelin and cholesterol metabolism may contribute to early CAS development [34]. 3β-Hydroxy-5-cholestenoate, an intermediate in bile acid synthesis, may reflect dysregulated bile acid metabolism or altered microbial modulation of bile acid conversion, potentially affecting systemic cholesterol homeostasis and inflammatory signaling, although its direct effects in vascular cells remain to be elucidated. Taken together, these findings suggest that Thermococcaceae may act as a “metabolic modulator,” potentially influencing CAS development by perturbing key lipid and sterol networks.

From a functional perspective, sphingomyelins and sterols influenced by Thermococcaceae, together with glutamine and its metabolites regulated by Bin127, are all involved in critical biological processes such as energy metabolism, oxidative stress, and lipid homeostasis. Although these metabolites belong to different molecular classes, they may exert interconnected effects on vascular homeostasis, inflammatory regulation, and atherosclerosis risk, suggesting that gut microbes may contribute to disease onset and progression *via* multiple, potentially complementary or synergistic metabolic pathways.

Although several associations identified in this study exhibited modest effect sizes, such magnitudes are common in complex multifactorial diseases such as CAS. The development of CAS is typically influenced by the cumulative effects of multiple genetic, metabolic, microbial, and environmental factors rather than a single dominant determinant [35]. Therefore, even moderate causal estimates may provide informative insights, particularly when the exposures are relatively prevalent in the general population. From a population health perspective, modest causal effects could still contribute to a meaningful attributable disease burden. In addition, the microbial taxa and metabolites identified in this study may serve as complementary molecular biomarkers, potentially aiding early risk identification and refinement of cardiometabolic risk stratification. In future translational studies, integrating these multi-omics features with established clinical risk models may support the exploration of more precise preventive strategies.

## 5 STUDY LIMITATIONS

However, several limitations should be noted. First, all GWAS datasets used in this study were derived from individuals of European ancestry, primarily from cohorts such as FinnGen. This limitation suggests that the generalizability of the findings to other populations may be limited, as gut microbiota composition and its interactions with host metabolism can be influenced by genetic background, environmental exposures, lifestyle, and dietary patterns. Moreover, diet-microbiome interactions and the genetic architecture underlying microbial traits and cardiometabolic risk may differ across ancestral groups. Consequently, the causal relationships identified here may not be directly applicable to non-European populations. Future studies in more diverse ancestral cohorts are warranted to validate and extend these findings, thereby improving their translational relevance. Second, although this study covered a broad range of microbial taxa and metabolites, it remains incomplete and may not fully capture the complexity of the gut microbial ecosystem and its metabolic networks. Furthermore, while MR enhances causal inference, potential horizontal pleiotropy cannot be completely excluded. Future studies integrating experimental models and multi-center clinical cohorts will be necessary to further validate the underlying biological mechanisms.

Overall, this study proposes a potential pathway through which gut microbes may influence CAS risk *via* plasma metabolites. Compared with simple association analyses, the approach here considers both the direction and magnitude of mediation effects. The results provide initial mechanistic insights into how specific microbes might influence CAS risk and highlight the potential importance of the “gut microbiota-metabolite” axis, offering a preliminary reference for possible preventive or therapeutic strategies. It should be emphasized that these findings require further validation, and future longitudinal studies combining molecular profiles with clinical phenotypes may provide more robust evidence to support precision cardiovascular medicine.

## CONCLUSION

This Mendelian randomization-based study provides genetic evidence suggesting a potential causal link among gut microbiota, plasma metabolites, and CAS. We identified certain microbial taxa and metabolites that may directly influence CAS risk, with plasma metabolites potentially mediating the effects of gut microbiota on CAS. Notably, *Species Bin127* and *Family Thermococcaceae* may influence CAS risk by modulating glutamine degradation products, sphingomyelins, and 3β-hydroxy-5-cholestenoate, highlighting the potential role of the “gut microbiota-metabolite” axis in the development and progression of CAS. These findings contribute to a better understanding of the potential mechanisms underlying CAS and may provide reference points for exploring risk-stratifying biomarkers, preventive or therapeutic strategies, and possible directions for precision cardiovascular health management.

## Figures and Tables

**Fig. (1):**
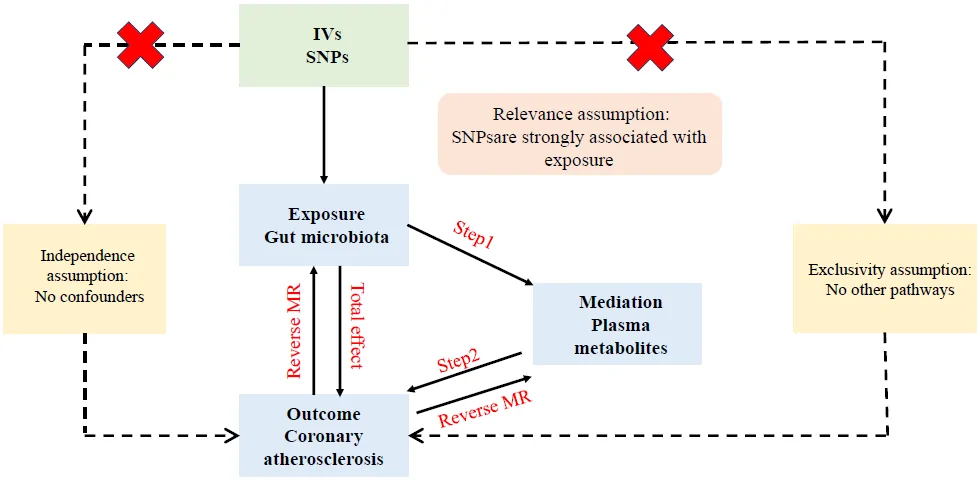
Flowchart of the Mendelian randomization study. IVs: Genetic variations linked to the exposure. Assumptions: The three key assumptions of Mendelian randomization analysis.

**Fig. (2):**
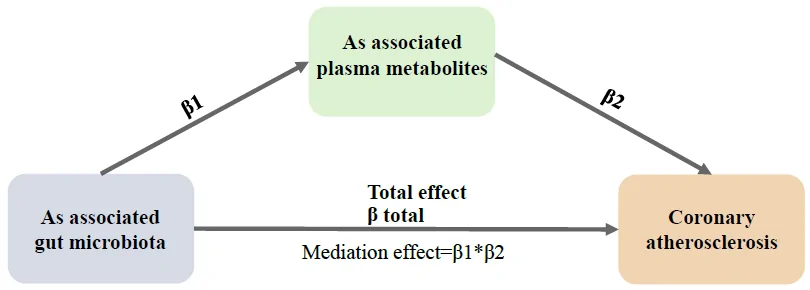
Flowchart of the mediation analysis. Total effect: The overall impact of gut microbiota exposure on coronary atherosclerosis. β1: The effect of gut microbiota on plasma metabolites. β2: The effect of plasma metabolites on coronary atherosclerosis.

**Fig. (3):**
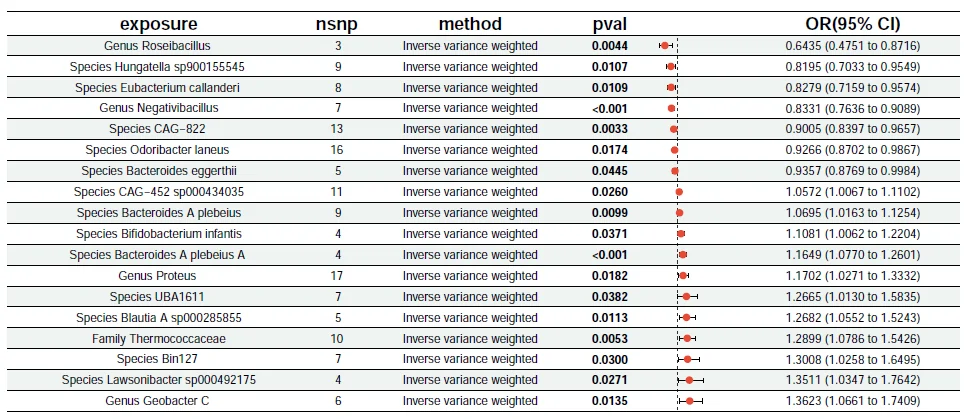
MR forest plot for gut microbiota and coronary atherosclerosis. Mendelian Randomization (MR) analysis was used to calculate the Odds Ratio (OR), 95% Confidence Interval (CI), and *p-value* for the association between gut microbiota exposure and coronary atherosclerosis outcomes. A *p-value* < 0.05 was considered statistically significant, with OR > 1 indicating a harmful factor and OR < 1 indicating a protective factor. 'nsnp' refers to the number of single-nucleotide polymorphisms.

**Fig. (4):**
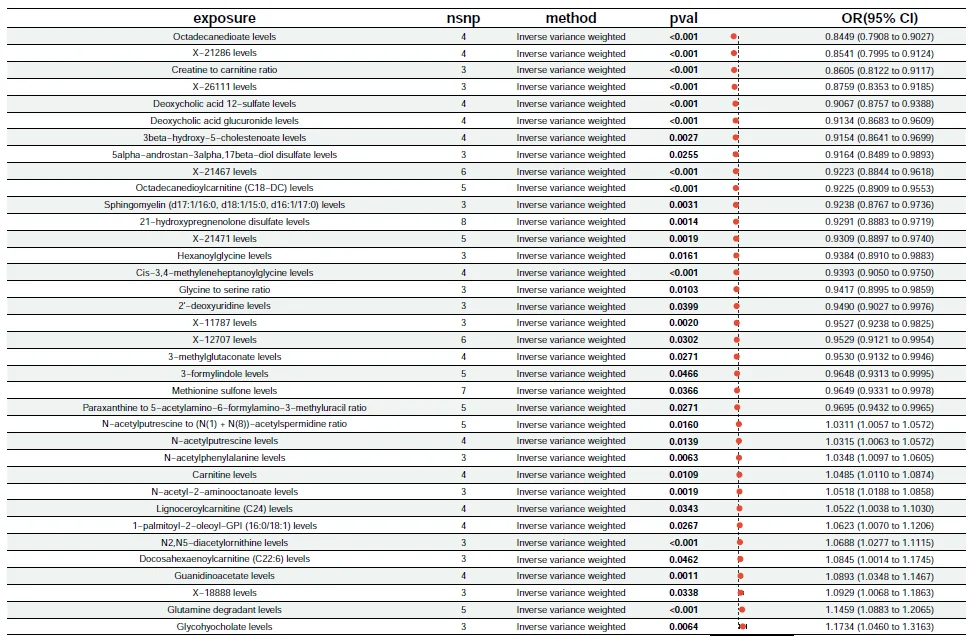
MR forest plot for plasma metabolites and coronary atherosclerosis. Mendelian Randomization (MR) analysis was used to calculate the odds ratio (OR), 95% Confidence Interval (CI), and *p-value* for the association between plasma metabolites and coronary atherosclerosis outcomes. A *p-value* < 0.05 was considered statistically significant, with OR > 1 indicating a harmful factor and OR < 1 indicating a protective factor. 'nsnp' refers to the number of single-nucleotide polymorphisms.

**Fig. (5):**
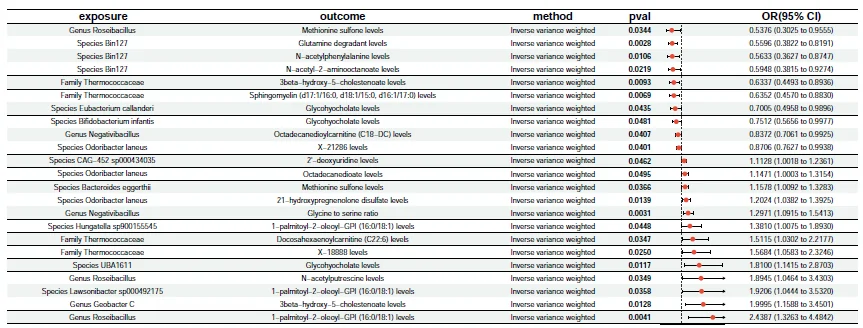
Mediation MR analysis of plasma metabolites. Mendelian Randomization (MR) was used to calculate the Odds Ratio (OR), 95% Confidence Interval (CI), and *p-value* for the association between gut microbiota and plasma metabolite exposure. A *p-value* < 0.05 was considered statistically significant, with OR > 1 indicating a harmful factor and OR < 1 indicating a protective factor. 'nsnp' refers to the number of single-nucleotide polymorphisms.

**Table 1:** Mediation effects and proportions of each plasma metabolite. Mediated effect: The influence of plasma metabolites in mediating the impact of gut microbiota on coronary atherosclerosis (with 95% confidence interval in parentheses). Mediated proportion: The percentage of the mediating effect (with 95% confidence interval in parentheses). Direct effect: The direct influence of gut microbiota exposure on coronary atherosclerosis outcomes. *P-value*: The significance level, with *p* < 0.05 considered statistically significant.

**Gut Microbiota**	**Plasma Metabolites**	**Outcome**	**Mediated Effect**	**Mediated Proportion**	**Direct Effect**	***p*-value**
*Species Bin127*	Glutamine degradant levels	CAS	-0.0791 (-0.139, -0.0192)	-30.1% (-52.8%, -7.28%)	0.342	0.0097
*Family Thermococcaceae*	Sphingomyelin (d17:1/16:0, d18:1/15:0, d16:1/17:0) levels	CAS	0.0359 (0.000642, 0.0712)	14.1% (0.252%, 28%)	0.219	0.0460
*Family Thermococcaceae*	3beta-hydroxy-5-cholestenoate levels	CAS	0.0403 (9.79e-05, 0.0805)	15.8% (0.0384%, 31.6%)	0.214	0.0494

## Data Availability

The data supporting the findings of this study are available from the corresponding author upon reasonable request.

## LIST OF ABBREVIATIONS

CAS: Coronary Atherosclerotic
MR: Mendelian Randomization
SNPs: Single-Nucleotide Polymorphisms
IVs: Instrumental Variables
GWAS: Genome-Wide Association Study
EA: Effect Allele
OA: Other Allele
EAF: Effect Allele Frequency
β: Effect Size
SE: Standard Error
ORs: Odds Ratios
CIs: Confidence Intervals
IVW: Inverse Variance Weighted
7-KC: 7-hydroxycholesterol
UC: Unesterified Cholesterol
LDL: Low-Density Lipoprotein
IMT: Intima-Media Thickness

## STANDARDS OF REPORTING

STROBE-MR guidelines and methodologies were followed.
