# Supplementary material for: Dissecting the Gut Microbiota-metabolite-coronary Atherosclerosis Axis: Evidence from Two-sample Mendelian Randomization
Source: Endocr Metab Immune Disord Drug Targets. 2026 Jul 21;26:E18715303456504. doi: 10.2174/0118715303456504260611042850 (PMC13635962; doi:10.2174/0118715303456504260611042850)
Supplement: Supplementary file 1 [file EMIDDT-26-E18715303456504_SD1.pdf]

Supplementary Material

Dissecting the Gut Microbiota-MetaboliteCoronary Atherosclerosis Axis: Evidence from Two-Sample Mendelian Randomization

Qitian Sun[1], Zeyu Zhou[2] and Yu Gao[1, 3,*]

[1]*Department of Endocrinology, Affiliated Hospital of Chengde Medical University, Chengde, Hebei, China;* [2]*Department of Clinical Laboratory, Affiliated Hospital of Chengde Medical University, Chengde, Hebei Province, China;* [3]*Hebei Key Laboratory of Panvascular Diseases, Chengde, Hebei Province, China*

exposure	outcome	nsnp	method	pval	OR(95% CI)
Species Bacteroides A plebeius A	Coronary atherosclerosis	4	MR Egger	0.2273	1.1811 (0.9772 to 1.4277)
		4	Weighted median	**0.0051**	1.1579 (1.0451 to 1.2830)
		4	Inverse variance weighted	**<0.001**	1.1649 (1.0770 to 1.2601)
		4	Simple mode	0.0936	1.1879 (1.0336 to 1.3651)
		4	Weighted mode	0.1672	1.1382 (0.9897 to 1.3091)
Species Bacteroides A plebeius	Coronary atherosclerosis	9	MR Egger	0.3765	1.0552 (0.9438 to 1.1797)
		9	Weighted median	0.0788	1.0615 (0.9932 to 1.1345)
		9	Inverse variance weighted	**0.0099**	1.0695 (1.0163 to 1.1254)
		9	Simple mode	0.3824	1.0511 (0.9457 to 1.1681)
		9	Weighted mode	0.3152	1.0531 (0.9580 to 1.1577)
Species Bacteroides eggerthii	Coronary atherosclerosis	5	MR Egger	0.0949	0.8253 (0.7061 to 0.9647)
		5	Weighted median	0.0616	0.9295 (0.8609 to 1.0036)
		5	Inverse variance weighted	**0.0445**	0.9357 (0.8769 to 0.9984)
		5	Simple mode	0.1803	0.9195 (0.8307 to 1.0177)
		5	Weighted mode	0.1825	0.9208 (0.8329 to 1.0181)
Species Bifidobacterium infantis	Coronary atherosclerosis	4	MR Egger	0.6093	1.1968 (0.6656 to 2.1518)
		4	Weighted median	0.0738	1.1060 (0.9903 to 1.2351)
		4	Inverse variance weighted	**0.0371**	1.1081 (1.0062 to 1.2204)
		4	Simple mode	0.2939	1.1032 (0.9480 to 1.2838)
		4	Weighted mode	0.2106	1.1052 (0.9768 to 1.2503)
Species Bin127	Coronary atherosclerosis	7	MR Egger	0.3271	1.7044 (0.6510 to 4.4625)
		7	Weighted median	0.0776	1.2983 (0.9715 to 1.7350)
		7	Inverse variance weighted	**0.0300**	1.3008 (1.0258 to 1.6495)
		7	Simple mode	0.2780	1.3442 (0.8267 to 2.1855)
		7	Weighted mode	0.2473	1.3497 (0.8532 to 2.1349)
Species Blautia A sp000285855	Coronary atherosclerosis	5	MR Egger	0.3301	1.7003 (0.6933 to 4.1701)
		5	Weighted median	0.0569	1.2804 (0.9927 to 1.6514)
		5	Inverse variance weighted	**0.0113**	1.2682 (1.0552 to 1.5243)
		5	Simple mode	0.4182	1.1790 (0.8243 to 1.6863)
		5	Weighted mode	0.4138	1.1729 (0.8323 to 1.6529)
Species CAG−452 sp000434035	Coronary atherosclerosis	11	MR Egger	0.4659	1.0651 (0.9056 to 1.2526)
		11	Weighted median	0.1790	1.0473 (0.9790 to 1.1205)
		11	Inverse variance weighted	**0.0260**	1.0572 (1.0067 to 1.1102)
		11	Simple mode	0.3922	1.0472 (0.9465 to 1.1586)
		11	Weighted mode	0.4068	1.0482 (0.9423 to 1.1660)
Species CAG−822	Coronary atherosclerosis	13	MR Egger	0.2018	0.8863 (0.7445 to 1.0551)
		13	Weighted median	0.0533	0.9094 (0.8259 to 1.0013)
		13	Inverse variance weighted	**0.0033**	0.9005 (0.8397 to 0.9657)
		13	Simple mode	0.2668	0.9100 (0.7765 to 1.0665)
		13	Weighted mode	0.2822	0.9100 (0.7722 to 1.0724)
Species Eubacterium callanderi	Coronary atherosclerosis	8	MR Egger	0.4501	0.7909 (0.4477 to 1.3974)
		8	Weighted median	0.0616	0.8376 (0.6956 to 1.0086)
		8	Inverse variance weighted	**0.0109**	0.8279 (0.7159 to 0.9574)
		8	Simple mode	0.3894	0.8756 (0.6593 to 1.1629)
		8	Weighted mode	0.4133	0.8834 (0.6680 to 1.1682)
Genus Geobacter C	Coronary atherosclerosis	6	MR Egger	0.0651	2.1755 (1.1897 to 3.9784)
		6	Weighted median	**0.0061**	1.5280 (1.1285 to 2.0690)
		6	Inverse variance weighted	**0.0135**	1.3623 (1.0661 to 1.7409)
		6	Simple mode	0.1107	1.5397 (0.9946 to 2.3837)
		6	Weighted mode	0.1181	1.5373 (0.9830 to 2.4040)
Species Hungatella sp900155545	Coronary atherosclerosis	9	MR Egger	0.5072	0.8412 (0.5178 to 1.3663)
		9	Weighted median	0.0989	0.8409 (0.6844 to 1.0331)
		9	Inverse variance weighted	**0.0107**	0.8195 (0.7033 to 0.9549)
		9	Simple mode	0.5687	0.9047 (0.6501 to 1.2589)
		9	Weighted mode	0.4099	0.8688 (0.6327 to 1.1929)
Species Lawsonibacter sp000492175	Coronary atherosclerosis	4	MR Egger	0.2914	2.0228 (0.7651 to 5.3479)
		4	Weighted median	0.0518	1.3732 (0.9976 to 1.8902)
		4	Inverse variance weighted	**0.0271**	1.3511 (1.0347 to 1.7642)
		4	Simple mode	0.1976	1.4464 (0.9329 to 2.2426)
		4	Weighted mode	0.1909	1.4352 (0.9424 to 2.1858)
Genus Negativibacillus	Coronary atherosclerosis	7	MR Egger	0.4068	0.8899 (0.6914 to 1.1455)
		7	Weighted median	**0.0134**	0.8629 (0.7677 to 0.9699)
		7	Inverse variance weighted	**<0.001**	0.8331 (0.7636 to 0.9089)
		7	Simple mode	0.1469	0.8630 (0.7255 to 1.0264)
		7	Weighted mode	0.1122	0.8685 (0.7486 to 1.0076)
Species Odoribacter laneus	Coronary atherosclerosis	16	MR Egger	0.5297	0.9587 (0.8434 to 1.0899)
		16	Weighted median	0.1145	0.9344 (0.8589 to 1.0165)
		16	Inverse variance weighted	**0.0174**	0.9266 (0.8702 to 0.9867)
		16	Simple mode	0.3248	0.9309 (0.8110 to 1.0685)
		16	Weighted mode	0.3257	0.9309 (0.8108 to 1.0688)
Genus Proteus	Coronary atherosclerosis	17	MR Egger	0.3512	1.1441 (0.8697 to 1.5052)
		17	Weighted median	0.1047	1.1442 (0.9724 to 1.3464)
		17	Inverse variance weighted	**0.0182**	1.1702 (1.0271 to 1.3332)
		17	Simple mode	0.0679	1.4242 (0.9996 to 2.0291)
		17	Weighted mode	0.2294	1.2503 (0.8808 to 1.7748)
Genus Roseibacillus	Coronary atherosclerosis	3	MR Egger	0.3208	0.4727 (0.2103 to 1.0624)
		3	Weighted median	**0.0148**	0.6139 (0.4146 to 0.9090)
		3	Inverse variance weighted	**0.0044**	0.6435 (0.4751 to 0.8716)
		3	Simple mode	0.1693	0.6013 (0.3748 to 0.9644)
		3	Weighted mode	0.1526	0.5953 (0.3795 to 0.9340)
Family Thermococcaceae	Coronary atherosclerosis	10	MR Egger	0.4257	1.2044 (0.7801 to 1.8596)
		10	Weighted median	**0.0112**	1.3834 (1.0767 to 1.7775)
		10	Inverse variance weighted	**0.0053**	1.2899 (1.0786 to 1.5426)
		10	Simple mode	0.0914	1.4662 (0.9859 to 2.1807)
		10	Weighted mode	0.1012	1.4702 (0.9720 to 2.2237)
Species UBA1611	Coronary atherosclerosis	7	MR Egger	0.4673	1.3154 (0.6642 to 2.6053)
		7	Weighted median	0.1090	1.2647 (0.9490 to 1.6855)
		7	Inverse variance weighted	**0.0382**	1.2665 (1.0130 to 1.5835)
		7	Simple mode	0.1789	1.3354 (0.9200 to 1.9383)
		7	Weighted mode	0.2372	1.3071 (0.8763 to 1.9496)

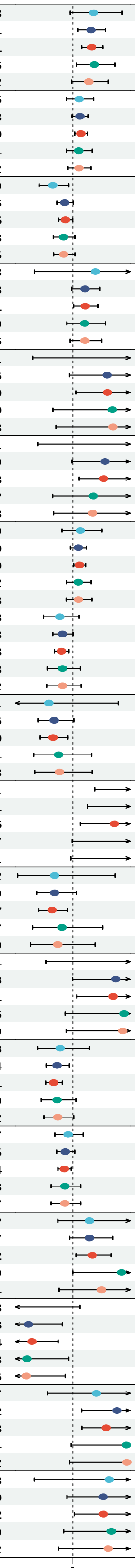

Fig. (S1).

exposure	outcome	nsnp	method	pval	OR(95% CI)
1−palmitoyl−2−oleoyl−GPI (16:0/18:1) levels	Coronary atherosclerosis	4	MR Egger	0.1758	1.2928 (1.0124 to 1.6510)
		4	Weighted median	0.1870	1.0465 (0.9782 to 1.1197)
		4	Inverse variance weighted	**0.0267**	1.0623 (1.0070 to 1.1206)
		4	Simple mode	0.7011	1.0219 (0.9242 to 1.1300)
		4	Weighted mode	0.5817	1.0256 (0.9463 to 1.1114)
2'−deoxyuridine levels	Coronary atherosclerosis	3	MR Egger	0.4053	0.9219 (0.8194 to 1.0372)
		3	Weighted median	**0.0472**	0.9490 (0.9011 to 0.9993)
		3	Inverse variance weighted	**0.0399**	0.9490 (0.9027 to 0.9976)
		3	Simple mode	0.6416	0.9790 (0.9069 to 1.0569)
		3	Weighted mode	0.1535	0.9416 (0.8936 to 0.9923)
21−hydroxypregnenolone disulfate levels	Coronary atherosclerosis	8	MR Egger	0.3574	0.9215 (0.7846 to 1.0822)
		8	Weighted median	**0.0047**	0.9264 (0.8785 to 0.9769)
		8	Inverse variance weighted	**0.0014**	0.9291 (0.8883 to 0.9719)
		8	Simple mode	0.9004	0.9936 (0.9017 to 1.0949)
		8	Weighted mode	**0.0414**	0.9040 (0.8350 to 0.9786)
3−formylindole levels	Coronary atherosclerosis	5	MR Egger	0.4243	0.9635 (0.8902 to 1.0427)
		5	Weighted median	**0.0076**	0.9531 (0.9201 to 0.9873)
		5	Inverse variance weighted	**0.0466**	0.9648 (0.9313 to 0.9995)
		5	Simple mode	0.4346	0.9652 (0.8911 to 1.0456)
		5	Weighted mode	0.0521	0.9484 (0.9130 to 0.9851)
3−methylglutaconate levels	Coronary atherosclerosis	4	MR Egger	0.2396	0.9259 (0.8453 to 1.0142)
		4	Weighted median	**0.0133**	0.9534 (0.9181 to 0.9901)
		4	Inverse variance weighted	**0.0271**	0.9530 (0.9132 to 0.9946)
		4	Simple mode	0.6952	0.9832 (0.9106 to 1.0617)
		4	Weighted mode	0.0758	0.9441 (0.9050 to 0.9848)
3beta−hydroxy−5−cholestenoate levels	Coronary atherosclerosis	4	MR Egger	0.6099	0.9552 (0.8222 to 1.1097)
		4	Weighted median	**<0.001**	0.9171 (0.8798 to 0.9559)
		4	Inverse variance weighted	**0.0027**	0.9154 (0.8641 to 0.9699)
		4	Simple mode	0.0948	0.8438 (0.7350 to 0.9687)
		4	Weighted mode	0.0855	0.9426 (0.9003 to 0.9868)
5alpha−androstan−3alpha,17beta−diol disulfate levels	Coronary atherosclerosis	3	MR Egger	0.2617	0.7632 (0.6059 to 0.9614)
		3	Weighted median	**0.0254**	0.9233 (0.8609 to 0.9902)
		3	Inverse variance weighted	**0.0255**	0.9164 (0.8489 to 0.9893)
		3	Simple mode	0.6900	0.9712 (0.8579 to 1.0996)
		3	Weighted mode	0.0817	0.8784 (0.8130 to 0.9492)
Carnitine levels	Coronary atherosclerosis	4	MR Egger	0.3610	1.0480 (0.9692 to 1.1332)
		4	Weighted median	**0.0078**	1.0535 (1.0138 to 1.0948)
		4	Inverse variance weighted	**0.0109**	1.0485 (1.0110 to 1.0874)
		4	Simple mode	0.3307	1.0468 (0.9688 to 1.1311)
		4	Weighted mode	0.0906	1.0565 (1.0113 to 1.1037)
Cis−3,4−methyleneheptanoylglycine levels	Coronary atherosclerosis	4	MR Egger	0.0848	0.9007 (0.8450 to 0.9600)
		4	Weighted median	**<0.001**	0.9298 (0.8969 to 0.9640)
		4	Inverse variance weighted	**<0.001**	0.9393 (0.9050 to 0.9750)
		4	Simple mode	0.2589	0.9442 (0.8707 to 1.0239)
		4	Weighted mode	**0.0271**	0.9256 (0.8917 to 0.9609)
Creatine to carnitine ratio	Coronary atherosclerosis	3	MR Egger	0.3428	0.8264 (0.6610 to 1.0331)
		3	Weighted median	**<0.001**	0.8598 (0.8062 to 0.9169)
		3	Inverse variance weighted	**<0.001**	0.8605 (0.8122 to 0.9117)
		3	Simple mode	0.0641	0.8507 (0.7819 to 0.9256)
		3	Weighted mode	0.0573	0.8576 (0.7953 to 0.9247)
Deoxycholic acid 12−sulfate levels	Coronary atherosclerosis	4	MR Egger	0.1324	0.8731 (0.7839 to 0.9724)
		4	Weighted median	**<0.001**	0.9003 (0.8656 to 0.9364)
		4	Inverse variance weighted	**<0.001**	0.9067 (0.8757 to 0.9388)
		4	Simple mode	**0.0468**	0.9029 (0.8493 to 0.9599)
		4	Weighted mode	**0.0183**	0.8987 (0.8594 to 0.9397)
Deoxycholic acid glucuronide levels	Coronary atherosclerosis	4	MR Egger	0.0621	0.8601 (0.7961 to 0.9292)
		4	Weighted median	**<0.001**	0.8947 (0.8568 to 0.9343)
		4	Inverse variance weighted	**<0.001**	0.9134 (0.8683 to 0.9609)
		4	Simple mode	0.1038	0.9050 (0.8315 to 0.9849)
		4	Weighted mode	**0.0129**	0.8955 (0.8599 to 0.9326)
Docosahexaenoylcarnitine (C22:6) levels	Coronary atherosclerosis	3	MR Egger	0.6809	1.1121 (0.7604 to 1.6265)
		3	Weighted median	0.0839	1.0864 (0.9890 to 1.1935)
		3	Inverse variance weighted	**0.0462**	1.0845 (1.0014 to 1.1745)
		3	Simple mode	0.2761	1.0897 (0.9728 to 1.2205)
		3	Weighted mode	0.2691	1.0889 (0.9753 to 1.2158)
Glutamine degradant levels	Coronary atherosclerosis	5	MR Egger	0.2130	1.1197 (0.9729 to 1.2887)
		5	Weighted median	**<0.001**	1.1597 (1.0833 to 1.2416)
		5	Inverse variance weighted	**<0.001**	1.1459 (1.0883 to 1.2065)
		5	Simple mode	**0.0357**	1.1629 (1.0576 to 1.2787)
		5	Weighted mode	**0.0293**	1.1664 (1.0652 to 1.2773)
Glycine to serine ratio	Coronary atherosclerosis	3	MR Egger	0.3101	0.9125 (0.8297 to 1.0035)
		3	Weighted median	**<0.001**	0.9346 (0.9066 to 0.9634)
		3	Inverse variance weighted	**0.0103**	0.9417 (0.8995 to 0.9859)
		3	Simple mode	0.0898	0.9267 (0.8832 to 0.9723)
		3	Weighted mode	**0.0485**	0.9340 (0.9059 to 0.9630)
Glycohyocholate levels	Coronary atherosclerosis	3	MR Egger	0.9009	1.0432 (0.6152 to 1.7688)
		3	Weighted median	0.0817	1.1093 (0.9870 to 1.2467)
		3	Inverse variance weighted	**0.0064**	1.1734 (1.0460 to 1.3163)
		3	Simple mode	0.3381	1.0982 (0.9480 to 1.2723)
		3	Weighted mode	0.3362	1.0976 (0.9491 to 1.2693)
Guanidinoacetate levels	Coronary atherosclerosis	4	MR Egger	0.7782	1.0217 (0.8964 to 1.1646)
		4	Weighted median	**0.0232**	1.0745 (1.0099 to 1.1432)
		4	Inverse variance weighted	**0.0011**	1.0893 (1.0348 to 1.1467)
		4	Simple mode	0.2271	1.0686 (0.9807 to 1.1645)
		4	Weighted mode	0.1642	1.0668 (0.9955 to 1.1431)
Hexanoylglycine levels	Coronary atherosclerosis	3	MR Egger	0.3168	0.6778 (0.4480 to 1.0255)
		3	Weighted median	**<0.001**	0.9170 (0.8778 to 0.9580)
		3	Inverse variance weighted	**0.0161**	0.9384 (0.8910 to 0.9883)
		3	Simple mode	0.1689	0.9149 (0.8424 to 0.9936)
		3	Weighted mode	0.0815	0.9109 (0.8615 to 0.9630)
Lignoceroylcarnitine (C24) levels	Coronary atherosclerosis	4	MR Egger	0.3247	1.1014 (0.9516 to 1.2747)
		4	Weighted median	**0.0088**	1.0582 (1.0143 to 1.1039)
		4	Inverse variance weighted	**0.0343**	1.0522 (1.0038 to 1.1030)
		4	Simple mode	0.0875	1.0891 (1.0187 to 1.1644)
		4	Weighted mode	0.1495	1.0479 (0.9992 to 1.0990)
Methionine sulfone levels	Coronary atherosclerosis	7	MR Egger	**0.0444**	0.9230 (0.8702 to 0.9790)
		7	Weighted median	**0.0054**	0.9497 (0.9158 to 0.9849)
		7	Inverse variance weighted	**0.0366**	0.9649 (0.9331 to 0.9978)
		7	Simple mode	0.3666	0.9628 (0.8922 to 1.0390)
		7	Weighted mode	**0.0312**	0.9457 (0.9095 to 0.9834)
N−acetyl−2−aminooctanoate levels	Coronary atherosclerosis	3	MR Egger	0.4667	1.0564 (0.9589 to 1.1639)
		3	Weighted median	**0.0018**	1.0493 (1.0180 to 1.0816)
		3	Inverse variance weighted	**0.0019**	1.0518 (1.0188 to 1.0858)
		3	Simple mode	0.4102	1.0316 (0.9725 to 1.0943)
		3	Weighted mode	0.1282	1.0447 (1.0097 to 1.0809)
N−acetylphenylalanine levels	Coronary atherosclerosis	3	MR Egger	0.7169	1.0110 (0.9664 to 1.0577)
		3	Weighted median	**0.0123**	1.0326 (1.0070 to 1.0588)
		3	Inverse variance weighted	**0.0063**	1.0348 (1.0097 to 1.0605)
		3	Simple mode	0.2194	1.0447 (0.9952 to 1.0967)
		3	Weighted mode	0.1675	1.0306 (1.0023 to 1.0596)
N−acetylputrescine levels	Coronary atherosclerosis	4	MR Egger	0.4524	1.0292 (0.9683 to 1.0940)
		4	Weighted median	**0.0169**	1.0314 (1.0056 to 1.0579)
		4	Inverse variance weighted	**0.0139**	1.0315 (1.0063 to 1.0572)
		4	Simple mode	0.5353	1.0177 (0.9688 to 1.0691)
		4	Weighted mode	0.0989	1.0318 (1.0054 to 1.0588)
N−acetylputrescine to (N(1) + N(8))−acetylspermidine ratio	Coronary atherosclerosis	5	MR Egger	0.1999	1.0377 (0.9928 to 1.0847)
		5	Weighted median	**0.0133**	1.0345 (1.0071 to 1.0627)
		5	Inverse variance weighted	**0.0160**	1.0311 (1.0057 to 1.0572)
		5	Simple mode	0.2930	1.0276 (0.9832 to 1.0741)
		5	Weighted mode	0.0648	1.0343 (1.0076 to 1.0617)
N2,N5−diacetylornithine levels	Coronary atherosclerosis	3	MR Egger	0.3781	1.0502 (0.9843 to 1.1206)
		3	Weighted median	**0.0014**	1.0666 (1.0252 to 1.1098)
		3	Inverse variance weighted	**<0.001**	1.0688 (1.0277 to 1.1115)
		3	Simple mode	0.1662	1.0888 (1.0070 to 1.1772)
		3	Weighted mode	0.0956	1.0646 (1.0219 to 1.1091)
Octadecanedioate levels	Coronary atherosclerosis	4	MR Egger	0.0785	0.7273 (0.6039 to 0.8760)
		4	Weighted median	**<0.001**	0.8215 (0.7660 to 0.8811)
		4	Inverse variance weighted	**<0.001**	0.8449 (0.7908 to 0.9027)
		4	Simple mode	**0.0377**	0.8224 (0.7386 to 0.9157)
		4	Weighted mode	**0.0131**	0.8205 (0.7625 to 0.8828)
Octadecanedioylcarnitine (C18−DC) levels	Coronary atherosclerosis	5	MR Egger	0.0963	0.9174 (0.8550 to 0.9845)
		5	Weighted median	**<0.001**	0.9208 (0.8917 to 0.9508)
		5	Inverse variance weighted	**<0.001**	0.9225 (0.8909 to 0.9553)
		5	Simple mode	0.1537	0.9428 (0.8828 to 1.0068)
		5	Weighted mode	**0.0139**	0.9240 (0.8904 to 0.9589)
Paraxanthine to 5−acetylamino−6−formylamino−3−methyluracil ratio	Coronary atherosclerosis	5	MR Egger	0.6175	0.9878 (0.9459 to 1.0316)
		5	Weighted median	**0.0053**	0.9726 (0.9537 to 0.9918)
		5	Inverse variance weighted	**0.0271**	0.9695 (0.9432 to 0.9965)
		5	Simple mode	0.5003	0.9807 (0.9312 to 1.0327)
		5	Weighted mode	0.0780	0.9751 (0.9549 to 0.9958)
Sphingomyelin (d17:1/16:0, d18:1/15:0, d16:1/17:0) levels	Coronary atherosclerosis	3	MR Egger	0.6697	0.9731 (0.8863 to 1.0685)
		3	Weighted median	**0.0083**	0.9274 (0.8769 to 0.9807)
		3	Inverse variance weighted	**0.0031**	0.9238 (0.8767 to 0.9736)
		3	Simple mode	0.1578	0.8722 (0.7725 to 0.9847)
		3	Weighted mode	0.1712	0.9388 (0.8849 to 0.9959)
X−11787 levels	Coronary atherosclerosis	3	MR Egger	0.3871	0.9523 (0.8910 to 1.0179)
		3	Weighted median	**0.0032**	0.9520 (0.9214 to 0.9837)
		3	Inverse variance weighted	**0.0020**	0.9527 (0.9238 to 0.9825)
		3	Simple mode	0.1206	0.9444 (0.9047 to 0.9858)
		3	Weighted mode	0.0949	0.9452 (0.9112 to 0.9806)
X−12707 levels	Coronary atherosclerosis	6	MR Egger	0.2447	0.9187 (0.8131 to 1.0379)
		6	Weighted median	**0.0164**	0.9387 (0.8914 to 0.9885)
		6	Inverse variance weighted	**0.0302**	0.9529 (0.9121 to 0.9954)
		6	Simple mode	0.2492	0.9428 (0.8630 to 1.0301)
		6	Weighted mode	0.0576	0.9387 (0.8925 to 0.9873)
X−18888 levels	Coronary atherosclerosis	3	MR Egger	0.3843	1.3070 (0.9102 to 1.8768)
		3	Weighted median	0.1082	1.0846 (0.9823 to 1.1975)
		3	Inverse variance weighted	**0.0338**	1.0929 (1.0068 to 1.1863)
		3	Simple mode	0.3405	1.0792 (0.9567 to 1.2174)
		3	Weighted mode	0.3333	1.0776 (0.9597 to 1.2100)
X−21286 levels	Coronary atherosclerosis	4	MR Egger	0.4786	0.8472 (0.5817 to 1.2340)
		4	Weighted median	**<0.001**	0.8613 (0.7927 to 0.9359)
		4	Inverse variance weighted	**<0.001**	0.8541 (0.7995 to 0.9124)
		4	Simple mode	0.1035	0.8829 (0.7946 to 0.9811)
		4	Weighted mode	0.0601	0.8627 (0.7821 to 0.9517)
X−21467 levels	Coronary atherosclerosis	6	MR Egger	0.3397	0.9577 (0.8856 to 1.0356)
		6	Weighted median	**<0.001**	0.9389 (0.9096 to 0.9691)
		6	Inverse variance weighted	**<0.001**	0.9223 (0.8844 to 0.9618)
		6	Simple mode	0.0773	0.9313 (0.8745 to 0.9918)
		6	Weighted mode	**0.0150**	0.9396 (0.9085 to 0.9717)
X−21471 levels	Coronary atherosclerosis	5	MR Egger	0.2073	0.8919 (0.7755 to 1.0258)
		5	Weighted median	**0.0239**	0.9503 (0.9091 to 0.9933)
		5	Inverse variance weighted	**0.0019**	0.9309 (0.8897 to 0.9740)
		5	Simple mode	0.2540	0.9650 (0.9158 to 1.0170)
		5	Weighted mode	0.2695	0.9644 (0.9123 to 1.0194)
X−26111 levels	Coronary atherosclerosis	3	MR Egger	0.2092	0.8510 (0.7640 to 0.9479)
		3	Weighted median	**<0.001**	0.8740 (0.8301 to 0.9203)
		3	Inverse variance weighted	**<0.001**	0.8759 (0.8353 to 0.9185)
		3	Simple mode	0.0635	0.8650 (0.8023 to 0.9326)
		3	Weighted mode	**0.0398**	0.8684 (0.8204 to 0.9192)

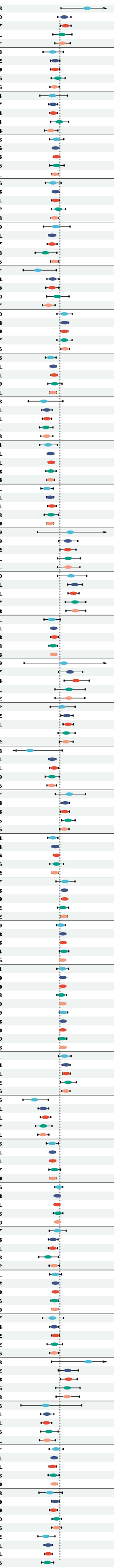

Fig. (S2)
